# New insights into the associations among feed efficiency, metabolizable efficiency traits and related QTL regions in broiler chickens

**DOI:** 10.1186/s40104-020-00469-8

**Published:** 2020-06-26

**Authors:** Wei Li, Ranran Liu, Maiqing Zheng, Furong Feng, Dawei Liu, Yuming Guo, Guiping Zhao, Jie Wen

**Affiliations:** 1 State Key Laboratory of Animal Nutrition; Key Laboratory of Animal (Poultry) Genetics Breeding and Reproduction, Ministry of Agriculture, Institute of Animal Sciences, Chinese Academy of Agricultural Sciences, Beijing, 100193 China; 2 College of Animal Science and Technology, China Agricultural University, Beijing, 100193 China; 3 Foshan Gaoming Xinguang Agricultural and animal Industrials Corporation, Foshan, 528515 China

**Keywords:** Broiler, Feed efficiency, Genome-wide association study, Imputation, Metabolizable efficiency

## Abstract

**Background:**

Improving the feed efficiency would increase profitability for producers while also reducing the environmental footprint of livestock production. This study was conducted to investigate the relationships among feed efficiency traits and metabolizable efficiency traits in 180 male broilers. Significant loci and genes affecting the metabolizable efficiency traits were explored with an imputation-based genome-wide association study. The traits measured or calculated comprised three growth traits, five feed efficiency related traits, and nine metabolizable efficiency traits.

**Results:**

The residual feed intake (RFI) showed moderate to high and positive phenotypic correlations with eight other traits measured, including average daily feed intake (ADFI), dry excreta weight (DEW), gross energy excretion (GEE), crude protein excretion (CPE), metabolizable dry matter (MDM), nitrogen corrected apparent metabolizable energy (AMEn), abdominal fat weight (AbF), and percentage of abdominal fat (AbP). Greater correlations were observed between growth traits and the feed conversion ratio (FCR) than RFI. In addition, the RFI, FCR, ADFI, DEW, GEE, CPE, MDM, AMEn, AbF, and AbP were lower in low-RFI birds than high-RFI birds (*P <* 0.01 or *P <* 0.05), whereas the coefficients of MDM and MCP of low-RFI birds were greater than those of high-RFI birds (*P <* 0.01). Five narrow QTLs for metabolizable efficiency traits were detected, including one 82.46-kb region for DEW and GEE on *Gallus gallus* chromosome (GGA) 26, one 120.13-kb region for MDM and AMEn on GGA1, one 691.25-kb region for the coefficients of MDM and AMEn on GGA5, one region for the coefficients of MDM and MCP on GGA2 (103.45–103.53 Mb), and one 690.50-kb region for the coefficient of MCP on GGA14. Linkage disequilibrium (LD) analysis indicated that the five regions contained high LD blocks, as well as the genes chromosome 26 C6orf106 homolog (*C26H6orf106*), LOC396098, SH3 and multiple ankyrin repeat domains 2 (*SHANK2*), ETS homologous factor (*EHF*), and histamine receptor H3-like (*HRH3L*), which are known to be involved in the regulation of neurodevelopment, cell proliferation and differentiation, and food intake.

**Conclusions:**

Selection for low RFI significantly decreased chicken feed intake, excreta output, and abdominal fat deposition, and increased nutrient digestibility without changing the weight gain. Five novel QTL regions involved in the control of metabolizable efficiency in chickens were identified. These results, combined through nutritional and genetic approaches, should facilitate novel insights into improving feed efficiency in poultry and other species.

## Introduction

Feed efficiency is the most important trait in the poultry industry because feed accounts for approximately 70% of the total production cost [1]. In poultry production, feed efficiency is generally defined as the relative ability of an animal to convert feed to product. The most widely used indexes for evaluating feed efficiency are the feed conversion ratio (FCR) and residual feed intake (RFI). FCR is the ratio between feed intake and body weight gain during the measurement period. RFI, which was first used by Koch et al. [2] for cattle, is generally defined as the difference between actual and expected feed intake, the latter of which is based on an animal’s requirements for maintaining body weight and for production [3]. Genetic selection for RFI has been reported to lead to reductions in true metabolizable energy intake and diet-induced thermogenesis in chickens [4]. Digestive efficiency is defined as the proportion of dietary intake minus feces, and metabolizable efficiency is defined as the proportion of dietary intake minus feces and urine [5]. In cattle, digestive efficiency is one of the five major physiological processes controlling RFI, and it conservatively explains 10% of the variation in RFI [6]. In poultry, metabolizable efficiency is easier to determine and is a more practical measure than digestive efficiency, because feces and urine are voided together via the single channel of the cloaca [5]. The heritability values of metabolizable efficiency, such as nitrogen corrected apparent metabolizable energy (AMEn), are moderate to high, ranging from 0.33 to 0.47 [7, 8]. Selection for metabolizable efficiency is accompanied by improved feed efficiency and reduced environmental impact [7, 9].

Quantitative trait loci (QTLs) for economically important traits in animals have been studied for more than 20 years. In chickens, 587 and 40 QTLs associated with FCR and RFI, respectively, have been detected in the Animal QTL Database (Animal QTLdb; https://www.animalgenome.org/cgi-bin/QTLdb/GG/index, 10/24/2019). To our knowledge, only three studies have reported 22 QTLs significantly associated with metabolizable efficiency traits, such as AMEn, dry excreta weight (DEW), and crude protein excretion (CPE) [8, 10, 11]. Few studies have been conducted in other chicken populations and most previous studies have been performed on two broiler lines divergently selected for low or high AMEn on a wheat-based diet.

A genome-wide association study (GWAS) is a powerful tool that can be used to explore the genomic variation associated with complex traits in farm animals. GWAS studies for RFI have been performed in broilers [12] and layers [13]. To date no GWAS for metabolizable efficiency traits has been reported in chickens. This study was conducted to clarify the relationships among feed efficiency traits and metabolizable efficiency traits, and identify significant loci and genes affecting metabolizable efficiency traits in fast-growing white-feathered broiler chickens.

## Materials and methods

### Experimental birds

**Fig. 1:**
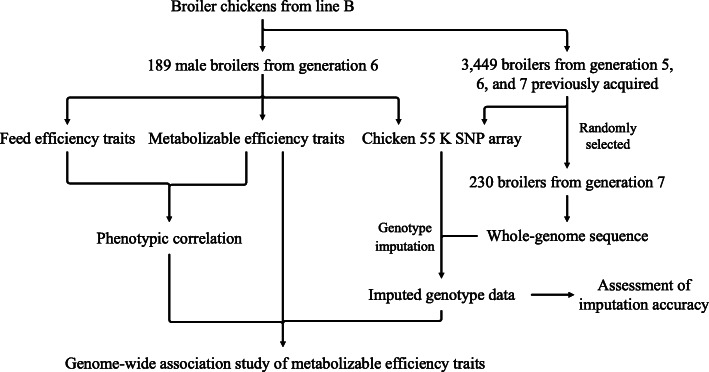
The workflow for the experiment in the current study

In the present study, the workflow for the experiment is illustrated in Fig. 1, and all chickens were obtained from the fast-growing white-feathered pure line B. Line B is a synthetic line produced by Foshan Gaoming Xinguang Agricultural and Animal Industrials Co., Ltd. (Foshan, China), and it has been selected for high body weight and growth rate traits for seven generations. In generation 6, a total of 189 male broiler breeders at 24 d of age, which were produced in the same hatch from 68 sires and 127 dams in generation 5, were randomly selected. They were housed in identical individual cages and provided with water and feed ad libitum. Each day, the amount of fresh feed provided was recorded individually, and residual feed was recorded daily and removed for an intervening period at 28 d of age. The broilers were fed a common corn-soybean meal diet until the end of the trial (42 d of age). The diet contained 2,900 kcal/kg metabolic energy and 183 g/kg crude protein, and detailed information about the diet is summarized in Table 1**.** The birds were slaughtered at 43 d of age after a 12-h overnight fast to obtain records of carcass traits, including abdominal fat weight (AbF).

**Table 1:** Ingredient and nutrient composition of the experimental diet

Ingredients, %		Nutrients composition	
Corn	67.35	Metabolizable energy, kcal/kg	2,900
Soybean meal	28.00	Crude protein, %	18.30
Soybean oil	0.40	Arginine, %	1.14
Limestone	1.90	Lysine, %	1.07
Monocalcium phosphate	1.00	Methionine, %	0.47
Salt	0.40	Methionine + Cystine, %	0.77
Lysine	0.22	Threonine, %	0.73
Methionine	0.17	Tryptophan, %	0.24
Choline chloride	0.14	Calcium, %	1.01
Premix^1^	0.42	Available phosphorus, %	0.31
Total	100		

^1^Premix supplied per kilogram of diet: vitamin A, 13,200 IU; vitamin D_3_, 5,000 IU; vitamin E, 60 IU; vitamin K_3_, 8.0 mg; thiamine, 4.0 mg; riboflavin, 12.0 mg; pyridoxine, 12.0 mg; cobalamin, 0.4 mg; nicotinic acid, 80 mg; pantothenic acid, 24 mg; folic acid, 2.0 mg; biotin, 3.0 mg; iron, 70.0 mg; copper, 8.5 mg; zinc, 45.0 mg; manganese, 60.0 mg; iodine, 0.85 mg; selenium, 0.20 mg; cobalt, 0.25 mg

### Metabolism trial and chemical analysis

A metabolism trial was conducted on 189 male broilers subjected to an adaptation period of 4 d followed by a subsequent collection period from 28 to 42 d of age. A total excreta collection method was used. During the collection period, the individual body weight and total feed intake were measured, and the daily (24 h) excreta was collected in white enamel trays. Contaminating materials (e.g., feathers, scales, and debris) were carefully removed. Then a small amount of 10% hydrochloric acid solution was sprayed to prevent nitrogen loss [14]. Excreta were collected daily for a fixed period of time and immediately placed in forced-air ventilation ovens at 65 °C for 24 h.

At the end of the collection period, dried excreta were weighted, ground through a 40-mesh screen, and stored in airtight plastic bags at − 20 °C for dry matter (DM), gross energy (GE), and crude protein (CP) analyses. The moisture content of the feed and excreta was removed by drying in forced-ventilation ovens at 105 °C until a constant weight was reached (AOAC method 934.01) [15]. GE values were determined through bomb calorimetry with a Parr 6400 adiabatic calorimeter (Parr Instrument Company, Illinois, USA), with benzoic acid used as the standard. CP content in samples was determined with the Kjeldahl method (AOAC method 984.13) using a Foss Kjeltec 2300 semiautomatic analyzer (Foss Tecator AB, Höganäs, Sweden) [15].

### Phenotypic measurements

Seventeen traits were measured or calculated: body weight at 28 d (BW28), BW42, average daily feed intake (ADFI), average daily gain (ADG), FCR, RFI, DEW, gross energy excretion (GEE), CPE, metabolizable dry matter (MDM), AMEn, metabolizable crude protein (MCP), coefficients of MDM, AMEn, and MCP, AbF, and percentage of abdominal fat (AbP). The total feed intake for each broiler was calculated by summing the feed consumption during the test period, which was then used to calculate the ADFI. The individual body weight gain was calculated on the basis of BW28 and BW42, and was then used to derive the ADG. The FCR was obtained by using the total feed intake divided by the total weight gain. For AMEn, 8.22 kcal/g of nitrogen was used as the correction factor [16]. The coefficients of MDM, AMEn, and MCP were calculated as input in feed minus output in excreta, divided by input in feed and multiplied by 100. The AbP was calculated as the ratio of AbF to BW42. The metabolic body weight at mid-test (MWT) was calculated as the average of BW28 and BW42 (MBW), raised to the power of 0.75 (MBW^0.75^) for each bird. RFI was computed as the difference between the observed and predicted ADFI. The predicted ADFI was calculated as:

$$ \mathrm{ADFI}=\mu +{\beta}_1\mathrm{MWT}+{\beta}_2\mathrm{ADG}+e $$

where *μ* represents the intercept, MWT represents the metabolic body weight at mid-test. ADG represents the average daily gain, *β*_1_ and *β*_2_ represent partial regression coefficients, and *e* represents the residual. The estimated *e* was taken as the measure of RFI.

Quality control of phenotypes was applied to RFI, FCR, and the coefficients of MDM, AMEn, and MCP, and data that deviated more than three standard deviations from the mean were removed. Nine birds were excluded from the analysis, and the remaining 180 records were available for further analyses.

### Genotyping, imputation, and quality control

Genomic DNA was extracted from blood samples with the phenol-chloroform method. The 189 broilers were genotyped with the customized chicken 55 K SNP array from Beijing Compass Biotechnology Co., Ltd. (Beijing, China) [17]. To improve the accuracy of imputation, in the following procedure we introduced a larger genotyping data for 3,449 broilers (1,926 males and 1,523 females) from three generations (generation 5, 6, and 7) of line B using 55 K SNP arrays previously acquired by our group.

For the target panel, a total of 3,638 broilers (2,115 males and 1,523 females) were used for genotype imputation. Quality control criteria were applied to the target panel: individual call rate ≥ 90%, SNP call rate ≥ 90%, and minor allele frequency (MAF) ≥ 0.01. In addition, SNPs located on the sex chromosomes were removed. Ultimately, 41,856 autosome variants and 3,607 broilers (2,097 males and 1,510 females) remained for further analyses.

For the reference panel, 230 broilers (101 males and 129 females) in generation 7 were randomly selected from 3,449 birds and re-sequenced with 150 bp paired-end reads on an Illumina NovaSeq 6000 platform with an average depth of approximately 10×1 L coverage. The sequencing was performed by Zhejiang Annoroad Biotechnology Co., Ltd. (Zhejiang, China). Variant calling was performed according to a standardized bioinformatics pipeline for all samples [18, 19]. Specifically, clean sequencing data were aligned to the chicken reference genome (GRCg6a/galGal6; ftp://ftp.ncbi.nlm.nih.gov/genomes/all/GCF/000/002/315/GCF_000002315.6_GRCg6a/) with the Burrows-Wheeler Aligner (BWA)-MEM algorithm [20]. Then, PCR duplicates were removed and local indel realignment and base-quality score recalibration were performed with the Genome Analysis Toolkit (GATK version 3.5) [21]. Variant calling was then performed via the HaplotypeCaller in GVCF mode with joint genotyping on all samples. Finally, SNPs were filtered with the GATK VariantFiltration protocol. The filtering settings were as follows: variant confidence score (QUAL) < 30.0, QualByDepth (QD) < 2.0, ReadPosRankSum < − 8.0, total depth of coverage (DP) < 4.0, FisherStrand (FS) > 60.0. SNPs on *Gallus gallus* chromosome (GGA) 16 were omitted because there were fewer common SNPs between the target panel and the reference panel. The sex chromosomes were also removed. In addition, quality control of the reference panel was conducted with the criteria of individual call rate ≥ 90%, SNP call rate ≥ 90%, and MAF ≥ 0.01. After filtering, a total of 12,377,431 autosome variants remained for the 230 sequenced birds.

Genotype imputation of the 55 K genotypes of the broilers to the imputed whole-genome sequence (WGS) level was performed with Beagle 5.0 [22]. Before imputation, inconsistencies between the target panel and the reference panel were checked with conform-gt software (http://faculty.washington.edu/browning/conform-gt.html). Subsequently, 40,342 autosomal SNPs from the target panel were retained. One factor, the effective population size [23], affects the accuracy of genotype imputation, because it is much smaller in livestock than in humans [24]. The effect of this factor on imputation from 55 K SNP chip data to WGS data was investigated to achieve higher accuracy. The microchromosome GGA28 was selected to improve computational efficiency.

The reference panel was pre-phased with Beagle 5.0 (default settings) [22]. Then the imputation from 55 K to WGS level was also executed in Beagle 5.0 with default parameters, except for setting the effective population size to 61,500 instead of the default of 1 million (Additional file 1**:** Fig. S1). To assess the accuracy of imputation from the target panel to the reference panel, we assessed the genotype concordance rate and allelic R^2^ measures for each variant. The genotype concordance rate was calculated by comparing the imputed and real genotypes for the 230 birds analyzed with both panels. The allelic R^2^ was calculated as the estimated squared correlation of the imputed sequence genotype on the true sequence, which was given by Beagle 5.0. We applied strict post-imputation filtering criteria per SNP: allelic R^2^ ≥ 0.9 and MAF ≥ 0.05. Finally, 1,279,346 autosomal variants and 180 samples remained for the GWAS analyses (Additional file 2: Table S1).

### Estimation of phenotypic correlation and genome-wide association study

Pairwise phenotypic correlations of two feed efficiency traits (RFI and FCR) with metabolizable efficiency traits were analyzed in the Hmisc R package. To better understand the relationships between RFI and metabolizable efficiency traits, the lowest and highest 20% of the RFI (low-RFI and high-RFI) birds were selected from 180 chickens and compared with Tukey’s test in SAS 9.4 (SAS Institute, NC) [25]. *P <* 0.05 (*) or *P <* 0.01 (**) was considered significant.

The GWAS for metabolizable efficiency traits was performed using the univariate linear mixed model (LMM) implemented in GEMMA version 0.98.1 software (https://github.com/genetics-statistics/GEMMA/releases) [26]. The genotype was set as the fixed factor and the additive polygenic effect as the random effect. Except for the coefficients of MDM, AMEn, and MCP, BW28 was considered as a covariate in the LMM for other traits. The statistical model was as follows:

$$ y=\mathbf{W}\alpha +\mathrm{x}\beta +\mathbf{u}+\epsilon; \mathbf{u}\sim \mathrm{MV}{\mathrm{N}}_{\mathrm{n}}\ \left(0,\lambda {\tau}^{-1}\mathbf{K}\right),\epsilon \sim \mathrm{MV}{\mathrm{N}}_{\mathrm{n}}\ \left(0,{\tau}^{-1}{\mathbf{I}}_n\right), $$

where *y* represents the vector of phenotypic values; **W** represents the vector of covariates, including a column of 1 s; *α* represents the vector of the corresponding coefficients including the intercept; *x* represents the vector of marker genotypes; *β* represents the effect size of the marker; *u* represents the vector of random polygenic effects; *ϵ* represents the vector of errors; *τ*^−1^ represents the variance of the residual errors; *λ* represents the ratio between the two variance components; K represents the centered relatedness matrix estimated from 1,279,346 variants and I_*n*_ represents the identity matrix. MVN_*n*_ represents the *n*-dimensional multivariate normal distribution. The Wald test was used as a criterion to select SNPs associated with metabolizable efficiency traits.

The genome-wide significance was assessed using the simple M method [27], to infer effective independent tests. A total of 85,247 independent tests over the entire chromosomal SNPs were obtained, and then genome-wide significant and suggestive thresholds were set to 5.87e-7 (0.05/85,247) and 1.17e-5(1/85,247), respectively. Manhattan and Q-Q plots were constructed for each trait by the qqman package (https://cran.r-project.org/web/packages/ qqman/) in R (version 3.6.0). Linkage disequilibrium (LD) blocks of target regions were performed using the Haploview version 4.2 software [28]. SNP positions were updated according to the newest release from UCSC (GRCg6a/galGal6 genome version). Identification of the closest genes to genome-wide significant and suggestive variants was obtained using UCSC annotation of the GRCg6a/galGal6 genome version (http://genome-asia.ucsc.edu/cgi-bin/hgGateway?hgsid=472768848_otkBtCHKhHMTV1xrxHuq737iivJ1). Boxplots were produced by the ggplot2 package in R (version 3.6.0).

## Results

### Descriptive statistics of traits

We determined the descriptive statistics for the traits associated with growth, feed efficiency, and metabolizable efficiency (Table 2). In this study, the ranges of RFI were − 16.72 g/d to 17.03 g/d, and the average FCR was 1.89 during the growing period from 28 to 42 d of age. The average BW28 was 1,254.67 g and reached 2,499.08 g of BW42. Birds digested an average of 167.95 g/d of feed, absorbed approximately 103.35 g/d of MDM, produced approximately 88.89 g/d of ADG, and excreted nearly 40.65 g/d of DEW. The coefficients of MDM, AMEn, and MCP averaged 71.80%, 71.42%, and 60.81%, respectively. The coefficients of variation of these traits in the population ranged from 1.36% to 33.96%.

**Table 2:** Descriptive statistics for growth, feed efficiency, and metabolizable efficiency traits of broilers

Traits^1^	*N*	Mean	SD	Min	Max	CV, %
RFI, g/d	180	0.00	5.69	−16.72	17.03	–
FCR, g:g	180	1.89	0.09	1.62	2.23	4.59
ADFI, g/d	180	167.95	14.46	119.07	198.36	8.61
BW28, g	180	1254.67	91.02	970.00	1530.00	7.25
BW42, g	180	2499.08	166.43	2020.00	2855.00	6.66
ADG, g/d	180	88.89	8.55	61.43	107.86	9.61
DEW, g/d	180	40.65	3.84	29.24	50.20	9.45
GEE, kcal/d	180	155.63	14.84	110.07	196.19	9.54
CPE, g/d	180	11.90	1.40	8.33	16.41	11.78
MDM, g/d	180	103.35	9.04	72.84	123.29	8.75
AMEn, kcal/d	180	448.94	39.37	317.20	532.27	8.77
MCP, g/d	180	18.38	1.64	12.84	22.82	8.91
Coefficient of MDM, %	180	71.80	1.09	68.67	75.71	1.52
Coefficient of AMEn, %	180	71.42	0.97	68.34	74.57	1.36
Coefficient of MCP, %	180	60.81	2.57	53.15	67.12	4.23
AbF, g	180	30.66	10.41	7.70	61.90	33.96
AbP, %	180	1.22	0.39	0.38	2.27	32.12

^1^*RFI* residual feed intake; *FCR* feed conversion ratio; *ADFI* average daily feed intake; *BW28* body weight at 28 d of age; *BW42* body weight at 42 d of age; *ADG* average daily gain; *DEW* dry excreta weight; *GEE* gross energy excretion; *CPE* crude protein excretion; *MDM* metabolizable dry matter; *AMEn* nitrogen corrected apparent metabolizable energy; *MCP* metabolizable crude protein; *AbF* weight of abdominal fat; *AbP* percentage of abdominal fat; *CV* coefficient of variation

### Phenotypic correlation analysis

Pearson correlation coefficients for feed efficiency traits (RFI and FCR) with other traits are shown in Table 3 and Additional file 3: Table S2. The positive phenotypic correlations of RFI with FCR, ADFI, DEW, GEE, CPE, MDM, AMEn, AbF, and AbP were moderate to high, ranging from 0.29 to 0.76 (*P <* 0.01). RFI showed a low correlation with MCP (r = 0.18; *P <* 0.05) and the coefficient of MDM (r = − 0.17; *P <* 0.05). RFI was phenotypically independent of BW28, BW42, and ADG (r = 0.00; *P >* 0.05). FCR was positively correlated with BW28 (r = 0.28; *P <* 0.01) and CPE (r = 0.18; *P <* 0.05), and negatively correlated with BW42, ADG, and MCP, ranging from − 0.49 to − 0.20 (*P <* 0.01). The relationships of FCR with ADFI, DEW, GEE, MDM, AMEn, AbF, and AbP were close to zero (*P >* 0.05). The same negative relationships existed between the coefficient of MCP and the two feed efficiency traits (RFI and FCR). Poor phenotypic correlations were found between the coefficient of AMEn and the two traits (RFI and FCR) (*P >* 0.05).

**Table 3:** Pearson correlation coefficients between feed efficiency traits with growth and metabolizable efficiency traits

Traits^1^	RFI	FCR
FCR	0.76^**^	–
ADFI	0.39^**^	−0.06
BW28	0.00	0.28^**^
BW42	0.00	−0.20^**^
ADG	0.00	−0.49^**^
DEW	0.43^**^	0.00
GEE	0.39^**^	−0.03
CPE	0.52^**^	0.18^*^
MDM	0.36^**^	−0.08
AMEn	0.39^**^	−0.05
MCP	0.18^*^	−0.25^**^
Coefficient of MDM	−0.17^*^	−0.14
Coefficient of AMEn	−0.01	0.02
Coefficient of MCP	−0.41^**^	−0.41^**^
AbF	0.29^**^	0.05
AbP	0.31^**^	0.09

^1^*RFI* residual feed intake; *FCR* feed conversion ratio; *ADFI* average daily feed intake; *BW28* body weight at 28 d of age; *BW42* body weight at 42 d of age; *ADG* average daily gain; *DEW* dry excreta weight; *GEE* gross energy excretion; *CPE* crude protein excretion; *MDM* metabolizable dry matter; *AMEn* nitrogen corrected apparent metabolizable energy; *MCP* metabolizable crude protein; *AbF* weight of abdominal fat; *AbP* percentage of abdominal fat; *P < 0.05, **P < 0.01

### Phenotypic differences between the low- and high-RFI broilers

Descriptive statistics for growth, feed efficiency, and metabolizable efficiency traits compared between the high- and the low-RFI broiler chickens are presented in Table 4. No significant difference was observed between high-RFI birds and low-RFI birds for BW28, BW42, ADG, MCP, and the coefficient of AMEn (*P >* 0.05). RFI and FCR were significantly lower for low-RFI birds than for high-RFI birds (*P <* 0.01). ADFI, DEW, GEE, CPE, MDM, AMEn, and AbP were 7.79%, 9.70%, 8.59%, 14.09%, 7.03%, 7.73%, and 20.90% lower, respectively, in low-RFI birds than high-RFI birds (*P <* 0.01). AbF was 18.97% lower in low-RFI chickens than high-RFI chickens (*P* < 0.05). Interestingly, the coefficients of MDM and MCP were greater for low-RFI birds than for high-RFI birds (*P <* 0.01).

**Table 4:** Means (±SD) for growth, feed efficiency, and metabolizable efficiency traits compared between the high- and low-RFI broiler chickens^1^

Traits^2^	Low-RFI	High-RFI	Low-RFI /High-RFI, %^3^
RFI, g/d	−7.52 ± 2.71	8.17 ± 3.31^**^	− 192.04
FCR, g:g	1.81 ± 0.06	1.99 ± 0.08^**^	−9.05
ADFI, g/d	161.39 ± 13.13	175.02 ± 14.33^**^	−7.79
BW28, g	1261.39 ± 112.43	1245.28 ± 81.29	1.29
BW42, g	2513.06 ± 174.62	2481.94 ± 166.53	1.25
ADG, g/d	89.40 ± 7.31	88.33 ± 9.53	1.21
DEW, g/d	38.63 ± 3.26	42.78 ± 4.04^**^	−9.70
GEE, kcal/d	148.96 ± 12.91	162.95 ± 15.29^**^	−8.59
CPE, g/d	11.10 ± 1.19	12.92 ± 1.40^**^	−14.09
MDM, g/d	99.74 ± 8.31	107.28 ± 8.53^**^	−7.03
AMEn, kcal/d	431.56 ± 35.86	467.73 ± 37.64^**^	−7.73
MCP, g/d	18.00 ± 1.43	18.64 ± 1.51	−3.43
Coefficient of MDM, %	72.11 ± 0.86^**^	71.52 ± 0.89	0.82
Coefficient of AMEn, %	71.44 ± 0.80	71.40 ± 0.73	0.06
Coefficient of MCP, %	61.97 ± 1.93^**^	59.17 ± 2.21	4.73
AbF, g	27.03 ± 9.5	33.36 ± 11.4^*^	−18.97
AbP, %	1.06 ± 0.34	1.34 ± 0.43^**^	−20.90

^1^low-RFI from male broilers with the 20% lowest RFI, *n* = 36 and high-RFI with the 20% highest RFI, *n* = 36; *P < 0.05, **P < 0.01

^2^*RFI* residual feed intake; *FCR* feed conversion ratio; *ADFI* average daily feed intake; *BW28* body weight at 28 d of age; *BW42* body weight at 42 d of age; *ADG* average daily gain; *DEW* dry excreta weight; *GEE* gross energy excretion; CPE, crude protein excretion; *MDM* metabolizable dry matter; *AMEn* nitrogen corrected apparent metabolizable energy; *MCP* metabolizable crude protein; *AbF* weight of abdominal fat; *AbP* percentage of abdominal fat

^3^Relative difference between low-RFI and high-RFI calculated as 100×(low-RFI mean/high-RFI mean − 1)

### Imputation accuracy

The average imputation accuracies of different effective population sizes on GGA28 are shown in Additional file 1: Fig. S1. As the effective population size increased from 50 to the default of 1 million, the genotype concordance rate slightly increased first and then decreased. The effective population size was set as 61,500 to obtain the highest accuracy of imputation.

**Fig. 2:**
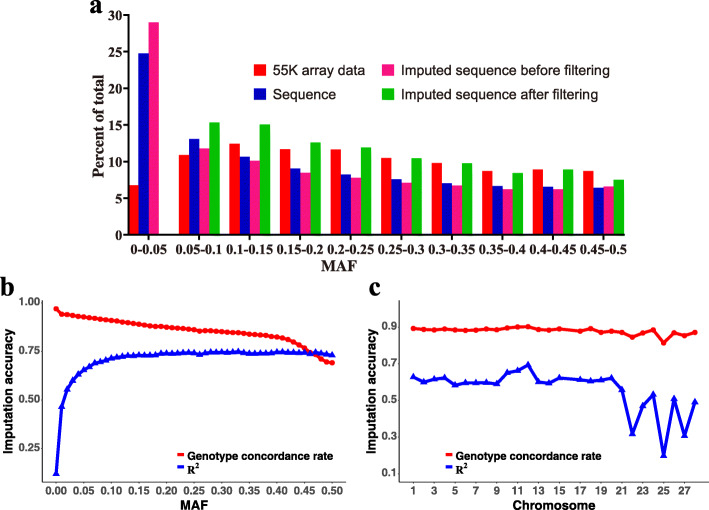
Distribution of MAF and imputation accuracy. **(a)** Percentage of SNPs in each MAF class for 55 K array data for 3607 birds, WGS data for 230 birds, and imputed WGS data for 3607 birds after imputation and post-imputation filtering. Post-imputation SNP filtering criteria: allelic R^2^ ≥ 0.9 and MAF ≥ 0.05. The imputation accuracy for imputed WGS data for 3607 birds after imputation according to MAF **(b)** and per chromosome **(c)** is also shown. MAF represents minor allele frequency

The number of SNPs in different MAF classes for different datasets are shown in Fig. 2a. In general, the MAF distribution from the four datasets showed the same trend, in which the number of SNPs per class slightly declined with increasing of MAF. Consistency was observed in the distribution of SNPs between 55 K array data and imputed WGS data after post-imputation filtering, and between WGS data and imputed WGS data after imputation (MAF ≥ 0.05). In addition, the MAF distribution based on 55 K array data was not significantly different from that based on WGS data (χ^2^-test, *P* = 0.15).

To evaluate the imputation accuracy for imputed WGS data for 3,607 birds after imputation in detail, we determined the average genotype concordance rate and R^2^ according to the MAF and chromosome, as shown in Fig. 2b, c. With increasing MAF, the genotype concordance rate decreased from 0.97 to approximately 0.69, whereas R^2^ increased from 0.12 to 0.75. At the chromosome level, the genotype concordance rate slightly fluctuated between 0.81 and 0.90, whereas R^2^ fluctuated between 0.20 and 0.69. The distribution of SNPs used in the GWAS analyses after post-imputation filtering is summarized in Additional file 2: Table S1. The genotypic concordance rate and R^2^ reached an average of 0.924 and 0.937, respectively.

### Genome-wide association study of metabolizable efficiency traits

**Fig. 3:**
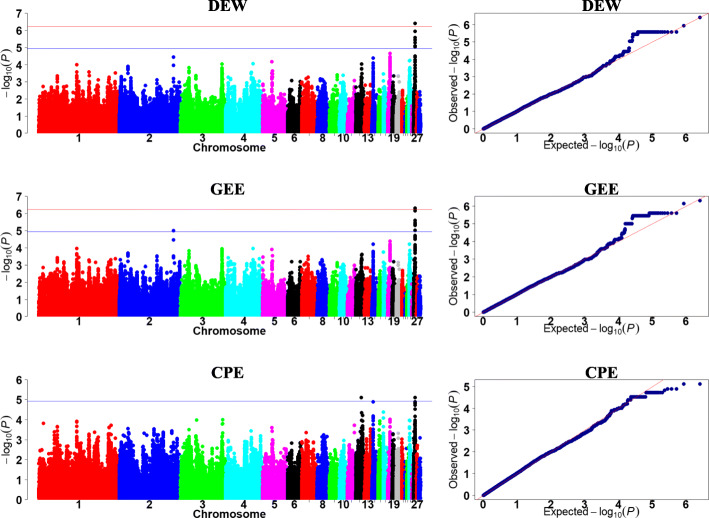
Manhattan and quantile-quantile plots of GWAS for excreta traits. Each dot represents a SNP in the dataset. The horizontal red and blue lines indicate the thresholds for genome-wide significance (*P* value = 5.87e-7) and suggestive significance (*P* value = 1.17e-5), respectively. DEW, GEE, and CPE represent dry excreta weight, gross energy excretion, and crude protein excretion, respectively

**Fig. 4:**
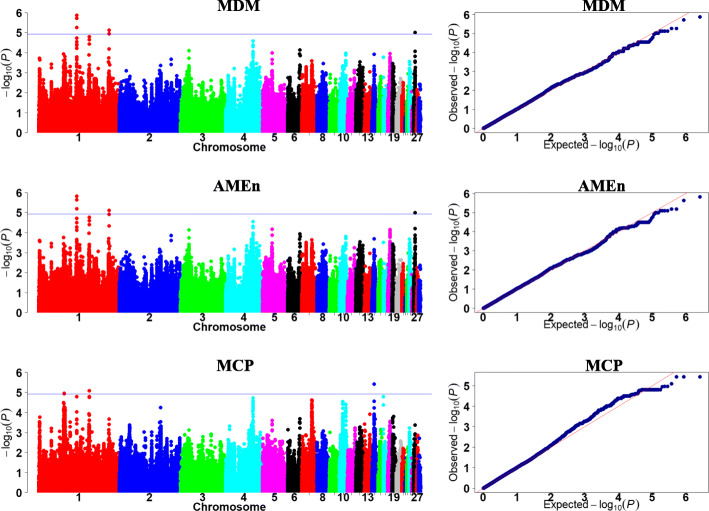
Manhattan and quantile-quantile plots of GWAS for metabolizable traits. Each dot represents a SNP in the dataset. The horizontal red and blue lines indicate the thresholds for genome-wide significance (*P* value = 5.87e-7) and suggestive significance (*P* value = 1.17e-5), respectively. MDM, AMEn, and MCP represent metabolizable dry matter, nitrogen corrected apparent metabolizable energy, and metabolizable crude protein, respectively

**Fig. 5:**
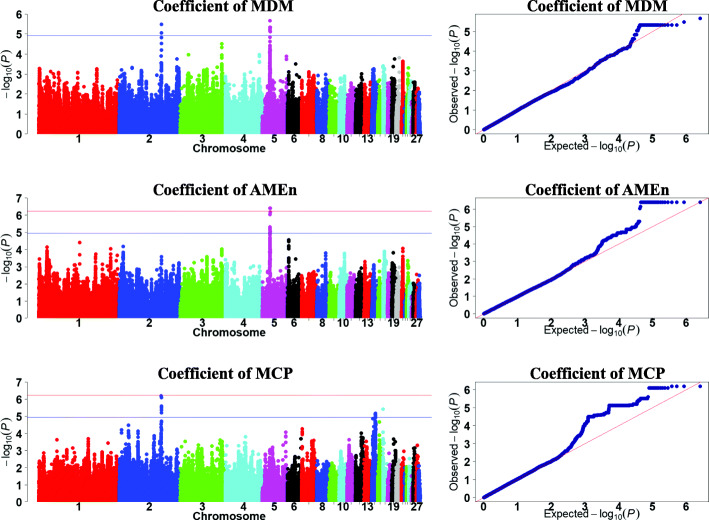
Manhattan and quantile-quantile plots of GWAS for the coefficients of metabolizable traits. Each dot represents a SNP in the dataset. The horizontal red and blue lines indicate the thresholds for genome-wide significance (*P* value = 5.87e-7) and suggestive significance (*P* value = 1.17e-5), respectively. The coefficients of MDM, AMEn, and MCP represent the coefficients of metabolizable dry matter, nitrogen corrected apparent metabolizable energy, and metabolizable crude protein, respectively

We focused on analysis of metabolizable efficiency traits in the current study. The Manhattan and quantile-quantile (Q-Q) plots are presented in Figs. 3, 4 and 5**,** and Table 5.

**Table 5:** Overview of the significant QTLs associated with metabolizable efficiency traits in broilers

Trait^a^	GGA^b^	Base-pair region		nSNP	Lead SNP (rsname)	Base pair	Alleles	MAF	*β*^c^	*P*-value	Candidate/nearest gene	Distance, kb^d^
		Start	End									
DEW	26	4,260,169	4,355,341	61	rs14300817	4,342,629	G/A	0.16	−2.66	3.91E-07	*C26H6orf106*	Exon 5
GEE	26	4,260,169	4,355,341	61	rs14300817	4,342,629	G/A	0.16	− 10.19	4.86E-07	*C26H6orf106*	Exon 5
MDM	1	93,262,479	93,382,604	4	rs732655996	93,382,604	C/T	0.07	−7.85	1.37E-06	*LOC396098*	D 35.93
AMEn	1	93,262,479	93,382,604	4	rs732655996	93,382,604	C/T	0.07	−34.04	1.53E-06	*LOC396098*	D 35.93
Coefficient of MDM	2	103,477,795	103,478,174	4	rs740788104	103,477,977	G/A	0.11	−0.77	3.27E-06	*HRH3L*	D 63.26
Coefficient of MDM	5	18,309,115	19,000,369	34	rs741135348	18,309,115	C/A	0.17	−0.73	2.17E-06	*SHANK2*	Intron 17
Coefficient of AMEn	5	18,309,115	19,000,369	53	rs315854959	18,992,840	A/G	0.19	−0.68	4.04E-07	*EHF*	U 5.96
Coefficient of MCP	2	103,454,037	103,528,655	45	rs15137100	103,454,067	T/C	0.08	−2.36	6.52E-07	*HRH3L*	D 39.35
Coefficient of MCP	14	10,016,503	10,707,002	206	rs738484580	10,067,812	G/A	0.13	−1.75	6.84E-06	NA	NA

^a^*DEW* dry excreta weight; *GEE* gross energy excretion; *MDM* metabolizable dry matter; *AMEn* nitrogen corrected apparent metabolizable energy; *MCP* metabolizable crude protein

^b^*Gallus gallus* chromosome

^c^Allele substitution effect was the additive effect estimated by GEMMA

^d^U and D indicate that the SNP is upstream and downstream of a gene, respectively

^e^NA represents not available

**Fig. 6:**
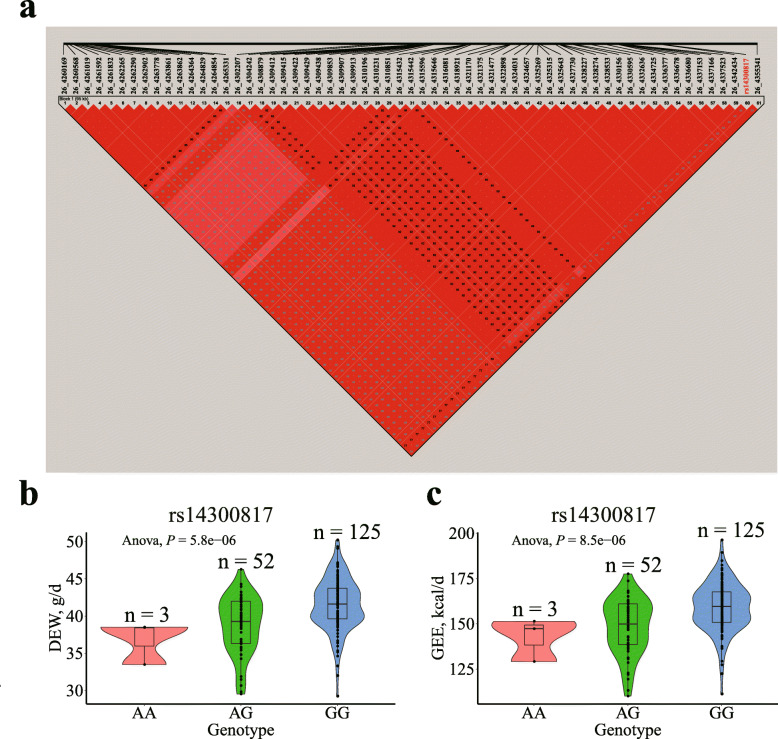
Association results of the candidate region on GGA26 (4.26–4.36 Mb) for DEW and GEE. **(a)** Linkage disequilibrium (LD) analysis of the 61 significant SNPs on GGA26. **(b)** Box plot for the effect of the SNP rs14300817 on DEW. **(c)** Box plot for the effect of the SNP rs14300817 on GEE. DEW and GEE represent dry excreta weight and gross energy excretion, respectively

For DEW and GEE, the same 95.17-kb region in GGA26 (4.26–4.36 Mb) was identified, which contained 61 significant SNPs. LD analysis showed that one high LD block was detected in this region (Fig. 6a). The most significant SNP in this region, rs14300817, had a negative effect (*β* < 0) of DEW and GEE, respectively. These SNPs on GGA26 were located either within or near the nearest genes, including chromosome 26 C6orf106 homolog (*C26H6orf106*).

For MDM and AMEn, four significant SNPs were clustered within a 120.13-kb region (GGA1: 93.26–93.38 Mb). The region with one strong block contained four significant SNPs (Additional file 4: Fig. S2a). The top SNP in this region, rs732655996, was located near a novel gene, LOC396098.

**Fig. 7:**
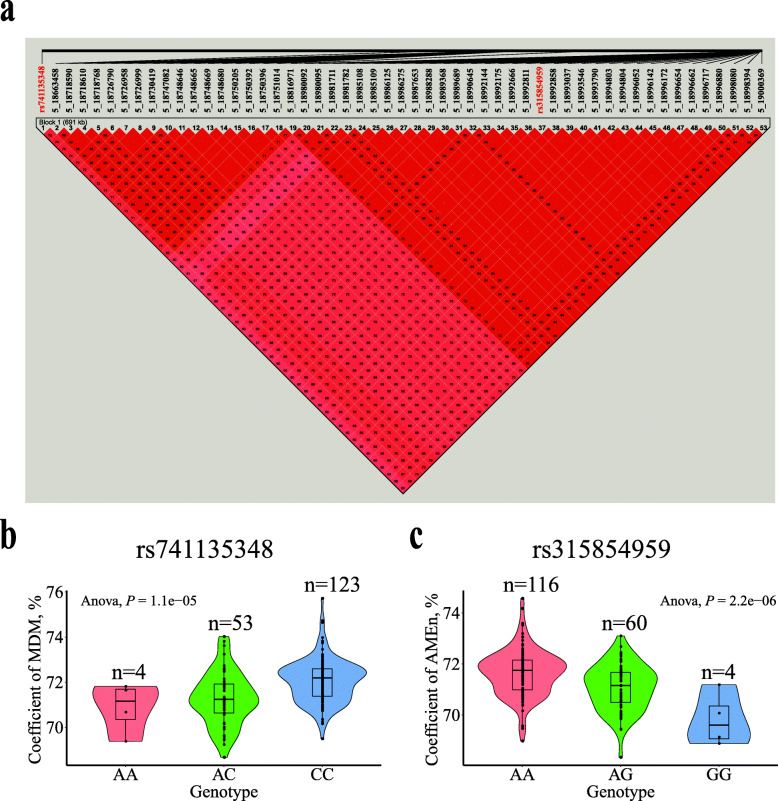
Association results of the candidate region on GGA5 (18.31–19.00 Mb) with the coefficients of MDM and AMEn. **(a)** Linkage disequilibrium (LD) analysis of the 53 significant SNPs on GGA5. **(b)** Box plot for the effect of the SNP rs741135348 on the coefficient of MDM. **(c)** Box plot for the effect of the SNP rs315854959 on the coefficient of AMEn. The coefficients of MDM and AMEn represent the coefficients of metabolizable dry matter and nitrogen corrected apparent metabolizable energy, respectively

For the coefficients of MDM and AMEn, a common 691.25-kb region (GGA5: 18.31–19.00 Mb) was detected, which contained 34 and 53 significant SNPs, respectively. LD analysis revealed one strong block in this significant region (Fig. 7a). The top SNP for the coefficient of MDM, rs741135348, was located in the 17th intron of SH3 and multiple ankyrin repeat domains 2 (*SHANK2*). The SNP rs315854959 was located near the ETS homologous factor (*EHF*). In addition, four significant SNPs associated with the coefficient of MDM were detected, which clustered within a 0.38-kb region in GGA2 (103.48 Mb). These SNPs were located near histamine receptor H3-like (*HRH3L*).

**Fig. 8:**
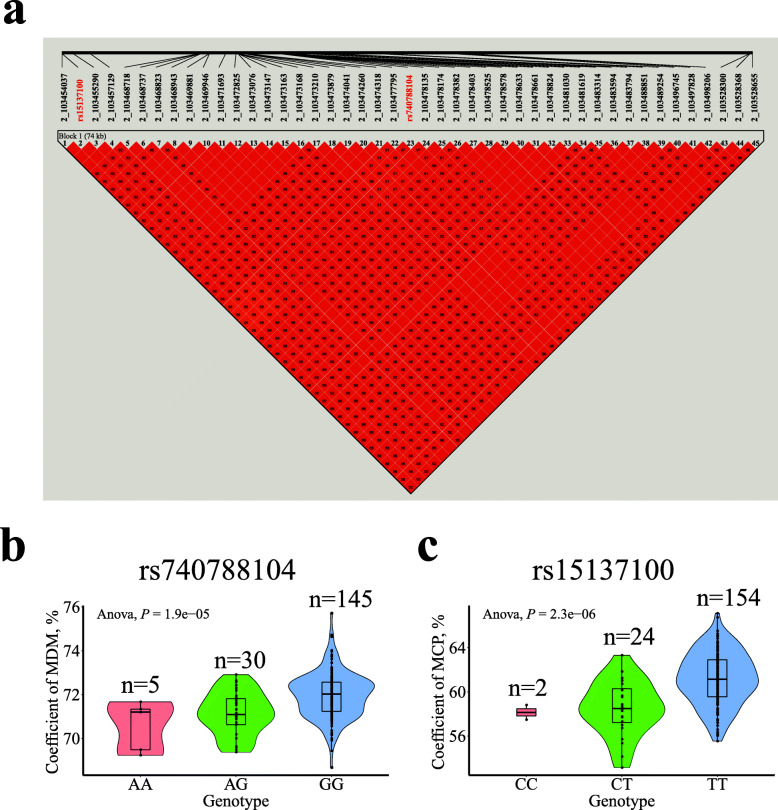
Association results of the candidate region on GGA2 (103.45–103.53 Mb) with the coefficients of MDM and MCP. **(a)** Linkage disequilibrium (LD) analysis of the 45 significant SNPs on GGA2. **(b)** Box plot for the effect of the SNP rs740788104 on the coefficient of MDM. **(c)** Box plot for the effect of the SNP rs15137100 on the coefficient of MCP. The coefficients of MDM and MCP represent the coefficients of metabolizable dry matter and metabolizable crude protein, respectively

For the coefficient of MCP, 251 significant SNPs were located in GGA2 and GGA14. The 45 significant SNPs were clustered within a 74.62-kb region (GGA2: 103.45–103.53 Mb), and four of these SNPs were the same as those found for the coefficient of MDM; 206 SNPs were clustered within a 690.50-kb region (GGA14: 10.02–10.71 Mb). Extremely strong LD status was found in the two regions (Fig. 8a and Additional file 5: Fig. S3a). The top variant on GGA2, rs15137100, was located near the *HRH3L* gene.

The effects of the most significant SNPs resulted in observed differences in the DEW, GEE, MDM, AMEn, and coefficients of MDM, AMEn, and MCP, as shown in Figs. 6, 7 and 8, and S2–S3. These results indicate that the lowest and highest phenotypic values belonged to homozygotes, whereas the intermediate values belonged to heterozygotes. Broilers with homozygous GG (rs14300817) excreted more DEW and GEE than did those with homozygous AA (Fig. 6b, c). MDM and AMEn were higher in the homozygous CC (rs732655996) than the homozygous TT broilers (Additional file 4: Fig. S2b–c). The coefficients of MDM, AMEn, and MCP were higher for broilers with homozygous CC (rs741135348), AA (rs315854959), GG (rs740788104), TT (rs15137100), and GG (rs738484580) than AA, GG, AA, CC, and AA (Figs. 7 and 8b–c, and Additional file 5: Fig. S3b).

## Discussion

### Phenotypic analysis

Metabolizable efficiency in classical animal nutrition can be used not only to evaluate the nutrient values of diets but also to directly account for individuals’ abilities to digest and absorb nutrients. For poultry, the index and total collection methods have been widely used for the determination of metabolizable efficiency. Although these methods involve laborious quantitative records of feed intake and output, the total collection method was chosen in the current study because it is more accurate, precise, and reproducible than the index method [5, 29]. To date there is no ideal inert indicator that has a uniform distribution in the diet and can be easily chemically determined [5]. In our study, feed intake and excreta were recorded for a 14-d test period consistent with poultry production, a period generally longer than those in previous studies, which have used a 2-d to 5-d period [10, 29, 30]. The coefficients of AMEn and MCP ranged from 68.34% to 74.57% (mean 71.42%) and from 53.15% to 67.12% (mean 60.81%), respectively, in agreement with findings by Wu et al. [31], who found that the coefficients of AMEn and MCP averaged 73% and 62% with ranges of 66–80% and 51–71%, respectively, in Ross 308 broilers fed 19 diets formulated with varying nutrient composition. Our results showed significant phenotypic correlations between growth traits (BW28, BW42, and ADG) and FCR in juvenile broilers, but not RFI, which have also been observed in slow-growing chickens [32] and ducks [33]. Therefore, selection for lower FCR focused on improving growth performance without decreasing feed intake.

Poultry excreta has always been of concern because it is associated with environmental pollution. Nutritional techniques such as reduction of dietary CP content have been used to decrease pollution, but these techniques have some undesirable effects on performance and appetite [34]. Therefore, genetic solutions should be determined. The heritability values of excretion traits from 17 to 23 d of age were low to moderate and ranged from 0.09 to 0.30 [9]. Tran et al. [10] detected nine QTLs significantly associated with metabolizable efficiency traits and selection for these traits should decrease excreta. In the current study, RFI presented a favorable phenotypic correlation with ADFI, DEW, GEE, CPE, MDM, and AMEn with moderate or high values, whereas weak correlations (almost zero) were observed between FCR and these traits. Different results for correlations between AMEn and the two traits (RFI and FCR) have been reported by Mignon-Grasteau et al. [7] and de Verdal et al. [30]; these studies used birds of the D+ and D- lines and found strongly negative genetic correlations between AMEn (kcal/kg) with FCR and RFI. Mignon-Grasteau et al. [35] estimated the heritability for AMEn on a corn-based diet and AMEn on a wheat-based diet to be 0.15 and 0.32, respectively, thereby indicating that the variability in the heritability of AMEn is largely influenced by diet composition. Thus, a reasonable explanation for the differences is that AMEn is an extremely complex trait affected by multiple factors including diet composition, test period, and calculation methods.

In addition, low-RFI birds had significantly lower ADFI, DEW, GEE, CPE, MDM, and AMEn, and higher the coefficients of MDM and MCP than the high-RFI birds. Similar results for low-RFI and high-RFI animals have been observed by Metzler-Zebeli et al. [36], who reported that low-RFI male broilers have lower DEW and CPE than high-RFI male broilers. Furthermore, Harris et al. [37] reported that low-RFI pigs have higher digestibility values for DM (87.3% vs. 85.9%), nitrogen (88.3% vs. 86.1%), and GE (86.9% vs. 85.4%) than high-RFI pigs. Mauch et al. [38] used divergent RFI lines and found that under a low-energy, high-fiber diet, low RFI pigs have greater digestibility of DM, GE, nitrogen, and neutral detergent fiber (7%, 7%, 10%, and 32%) than high RFI pigs (*P <* 0.05).

Excess AbF deposition is undesirable in the poultry industry because AbF is considered a waste product [39]. In the current study, AbF and AbP had moderate, positive phenotypic correlations with RFI but not with FCR. This is in agreement with findings from Wen et al. [32], in which abdominal fat had a moderate positive phenotypic correlation with RFI and not with FCR in slower growing chickens. In addition, we found that the low-RFI birds had significantly lower AbF and AbP than the high-RFI birds. This result suggests that selection for lower RFI animals could decrease fat deposition in growing animals.

### Genotype imputation

Genotype imputation has been widely used in GWAS to boost power [40]. This method can aid in identifying many novel SNPs and QTLs associated with phenotypes of interest. In previous GWAS-based studies, imputation from low density SNP chip genotypes to the WGS level had been implemented in chickens [41], pigs [42], and cattle [43]. Imputed genotypes with sufficiently high imputation accuracy are necessary for reliable results in follow-up analyses such as GWAS. In the current study, the genotypic concordance rate and R^2^ between imputed and true genotypes reached an average of 0.924 and 0.937, respectively, values that were higher than those reported by Huang et al. [41], who used sequence data imputed from a SNP array for GWAS in chickens and achieved an average imputation accuracy of 0.914. Hayes et al. [44] reported that the accuracy of mimic imputation from the 50 K panel to WGS was 83–93% in Beagle (version 3) for sheep breeds. Ni et al. [45] reported that the post-imputation filtering criterion for imputation accuracy should be 0.80 to ensure the high quality of the imputed WGS data.

### Genome-wide association study of metabolizable efficiency traits

#### *Loci* and genes for DEW, GEE, MDM, and AMEn

The genomic region of 95.17 kb on GGA26 (4.26–4.36 Mb) was detected to be associated with DEW and GEE. Previous studies have reported QTLs on GGA26 for DEW (2.4–3.2 Mb) and excreta nitrogen to phosphorus ratio (3.2–4.2 Mb) in an F_2_ resource population of medium-growth broilers [11]. The most significant SNP associated with DEW and GEE was located in the fifth exon of the *C26H6orf106* gene. Its homolog, *C6orf106*, has been investigated for activation of extracellular-signal-regulated kinase signaling pathways to accelerate cell proliferation [46].

A genomic region (GGA1: 93.26–93.38 Mb) was found to be associated with MDM and AMEn, and has also been found to be included in a feed efficiency QTL (GGA1: 90.35**–**123.03 Mb) in a meat-type × egg-type resource population by Hansen et al. [47]. The highly significant SNP is located in the unannotated gene LOC396098, which has no known function in chickens.

#### *Loci* and genes associated with the coefficients of MDM, AMEn, and MCP

We detected one important QTL region (GGA5: 18.31–19.00 Mb) associated with the coefficients of MDM and AMEn, which was not previously reported. The top SNPs associated with DEW and GEE were within the *SHANK2* and *EHF* genes. *SHANK2* (also known as ProSAP1) is the second member of the Shank protein family and is involved in neurodevelopmental and psychiatric disorders [48]. *SHANK2* plays a key role in regulating transepithelial salt and water transport by modulating Na^+^/H^+^ exchanger 3 (*NHE3*) expression and activity in epithelial cells, including those in the gastrointestinal tract [49]. *SHANK2*-knockout mice show hyperactivity and repetitive behaviors [50]. *EHF* is a member of the epithelium-specific ETS transcription factor family, which is highly expressed in multiple epithelial cell types including intestinal epithelium [51]. *EHF* plays an important role in the regulation of epidermal proliferation and differentiation [52].

Two novel regions (GGA2: 103.45–103.53 Mb and GGA14: 10.02–10.71 Mb) associated with the coefficient of MCP were detected and the top variant was located near the *HRH3L* gene. Very few studies on *HRH3L* are available in the literature. *HRH3* is an autoreceptor on numerous neurons that inhibits the synthesis and release of histamine [53, 54]. Previous studies have shown that *HRH3* negatively regulates food intake in rodents, in a manner independent of its histaminergic tone modulation [55].

## Conclusions

In summary, selection for low RFI significantly decreased chicken feed intake, excreta output, and abdominal fat deposition, and increased nutrient digestibility without changing weight gain. In addition, five novel QTL regions involved in the control of metabolizable efficiency in chickens were identified. These results, obtained from both nutritional and genetic approaches, should facilitate novel insights into improving feed efficiency in poultry and other species.

## Supplementary information

**Additional file 1: Figure S1.** Average imputation accuracies of different effective population sizes on GGA28.

**Additional file 2: Table S1.** Distribution of SNPs used in the GWAS analyses after post-imputation filtering.

**Additional file 3: Table S2.** Pearson correlation coefficients for growth and metabolizable traits in broilers.

**Additional file 4: Figure S2.** Association results of the candidate region on GGA1 (93.26–93.38 Mb) for MDM and AMEn. (a) Linkage disequilibrium (LD) analysis of the 4 significant SNPs on GGA1. (b) Box plot for the effect of the SNP rs732655996 on MDM. (c) Box plot for the effect of the SNP rs732655996 on AMEn. MDM and AMEn represent metabolizable dry matter and nitrogen corrected apparent metabolizable energy, respectively.

**Additional file 5: Figure S3.** Association results of the candidate region on GGA14 (10.02–10.71 Mb) for coefficient of MCP. (a) Linkage disequilibrium (LD) analysis of the 206 significant SNPs on GGA14. (b) Box plot for the effect of the SNP rs738484580 on the coefficient of MCP. Coefficient of MCP represents the coefficient of metabolizable crude protein.

## Data Availability

The raw whole genome sequencing data reported in this paper have been deposited in the Genome Sequence Archive [56] in BIG Data Center [57] under accession number CRA002454 that can be publicly accessed at https://bigd.big.ac.cn/gsa. The genotype and phenotype data of the 180 samples used in GWAS are available at the figshare repository (https://figshare.com/s/8c882576e0bc014fe382).

## Abbreviations

AbF: Weight of abdominal fat
AbP: Percentage of abdominal fat
ADFI: Average daily feed intake
ADG: Average daily gain
AMEn: Nitrogen corrected apparent metabolizable energy
BW28: Body weight at 28 d of age
BW42: Body weight at 42 d of age
C26H6orf106: Chromosome 26 C6orf106 homolog
CP: Crude protein
CPE: Crude protein excretion
CV: Coefficient of variation
d: Day
DEW: Dry excreta weight
DM: Dry matter
EHF: ETS homologous factor
FCR: Feed conversion ratio
GE: Gross energy
GEE: Gross energy excretion
GGA: *Gallus gallus* chromosome
GWAS: Genome-wide association study
HRH3L: Histamine receptor H3-like
LD: Linkage disequilibrium
MCP: Metabolizable crude protein
MDM: Metabolizable dry matter
MWT: Metabolic body weight at mid-test
QTL: Quantitative trait locus
Animal QTLdb: Animal QTL Database
SHANK2: SH3 and multiple ankyrin repeat domains 2
SNP: Single nucleotide polymorphism
WGS: Whole-genome sequence
RFI: Residual feed intake
