# Supplementary figures and images for: New insights into the associations among feed efficiency, metabolizable efficiency traits and related QTL regions in broiler chickens
Source: J Anim Sci Biotechnol. 2020 Jun 26;11:65. doi: 10.1186/s40104-020-00469-8 (PMC7318453; doi:10.1186/s40104-020-00469-8)

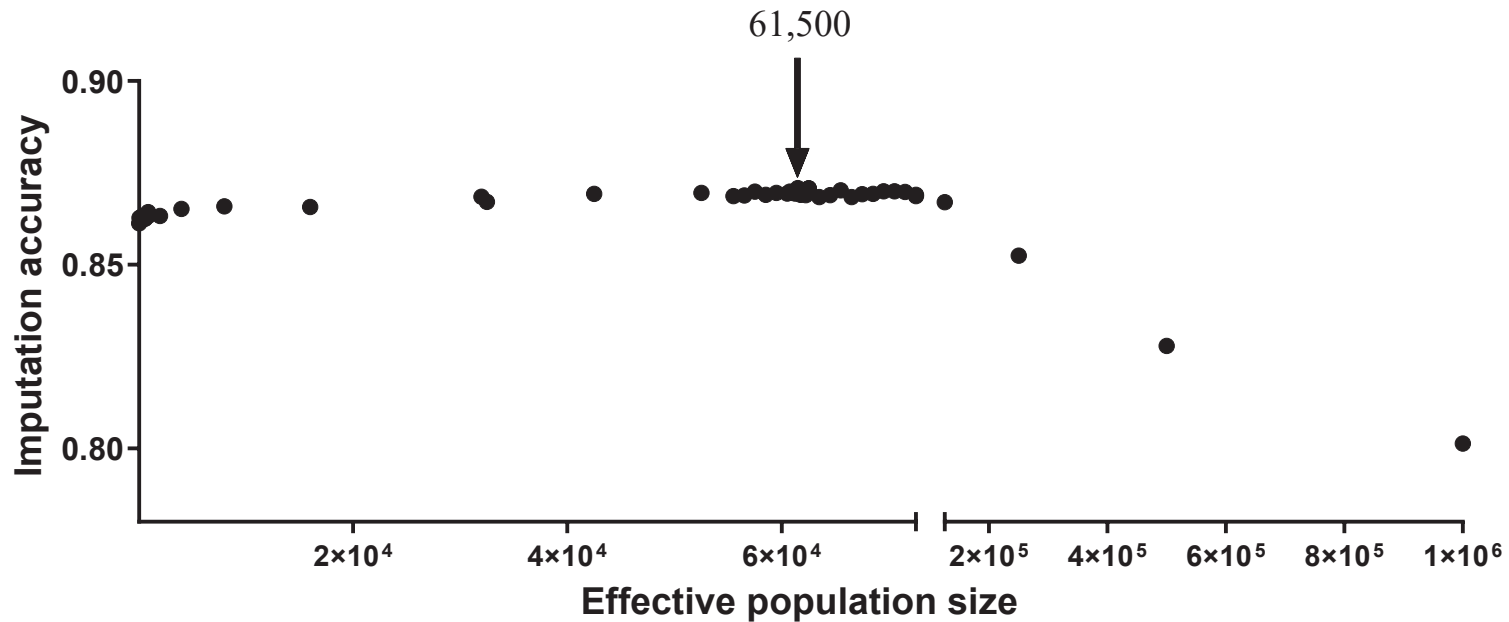

Supplement: Supplementary file 1 — Additional file 1: Figure S1. Average imputation accuracies of different effective population sizes on GGA28. [file 40104_2020_469_MOESM1_ESM.pdf]

**a**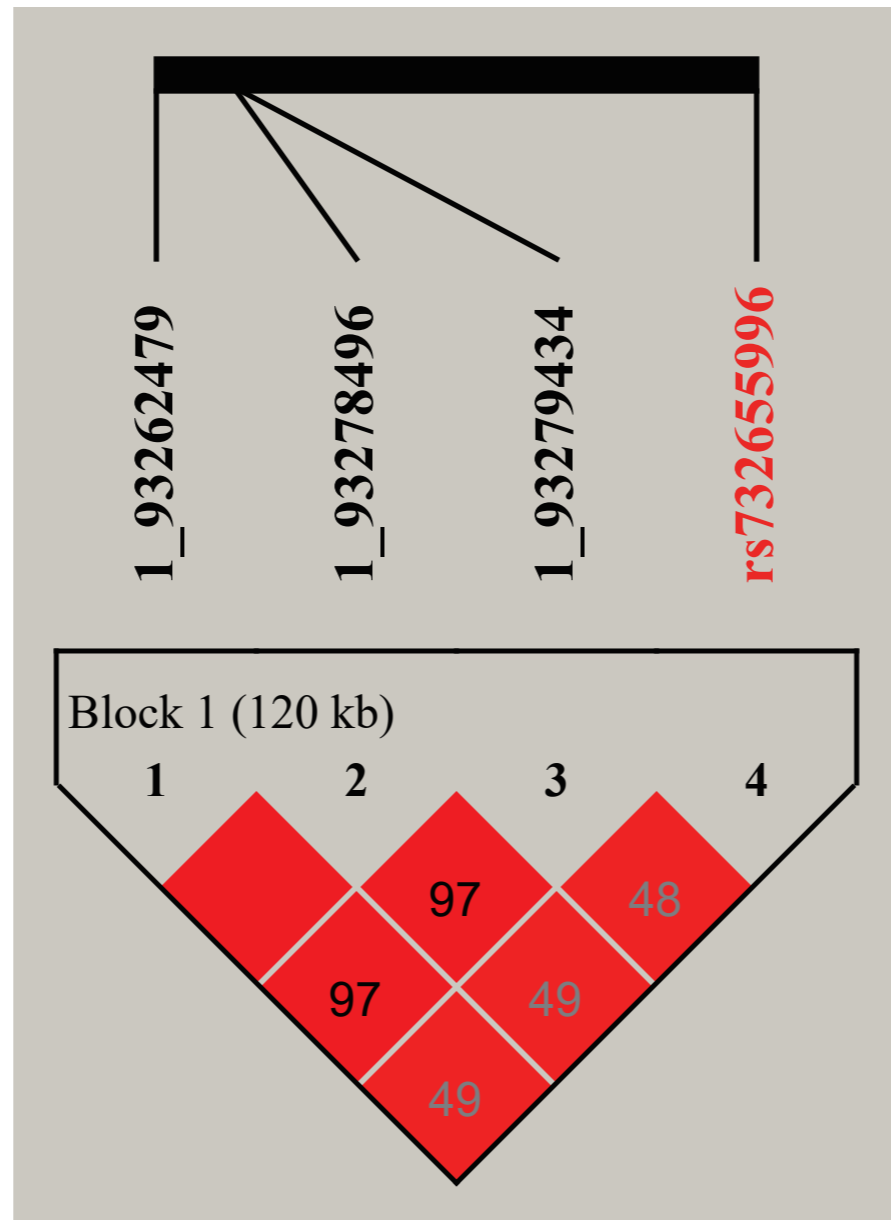**b**

rs732655996

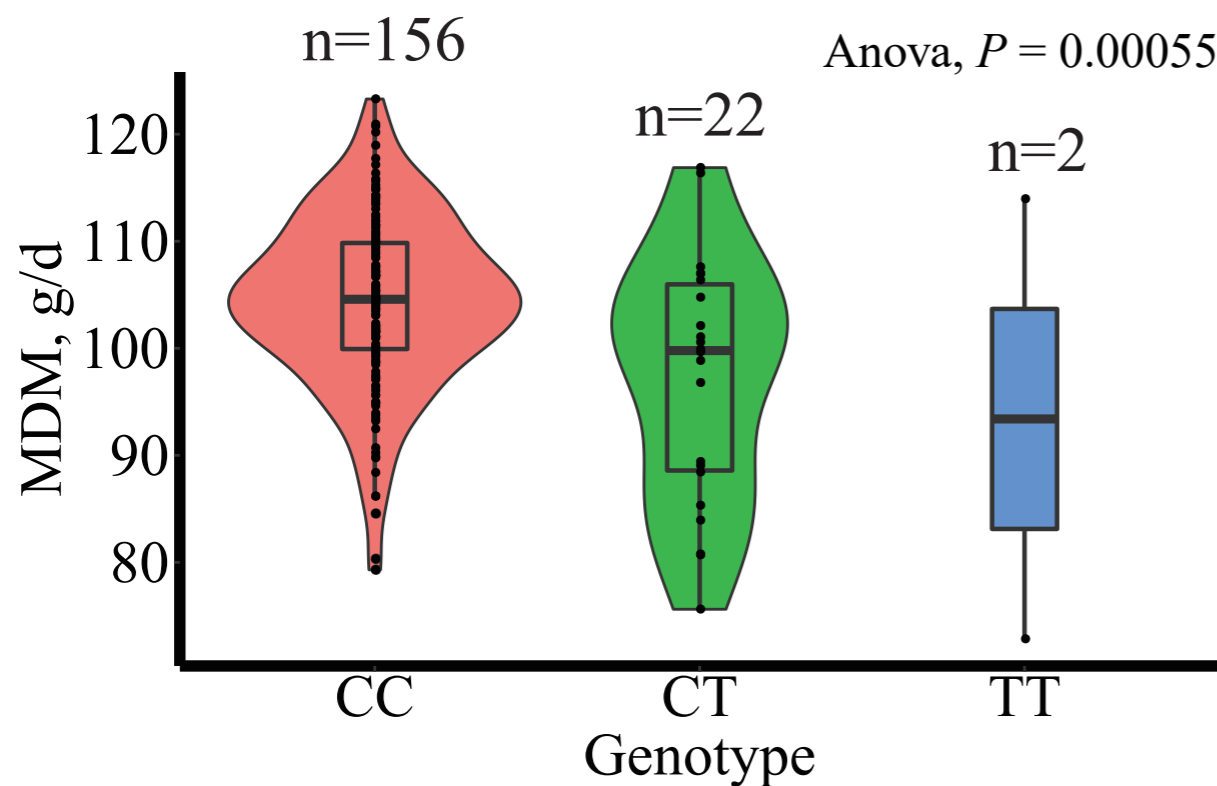**c**

rs732655996

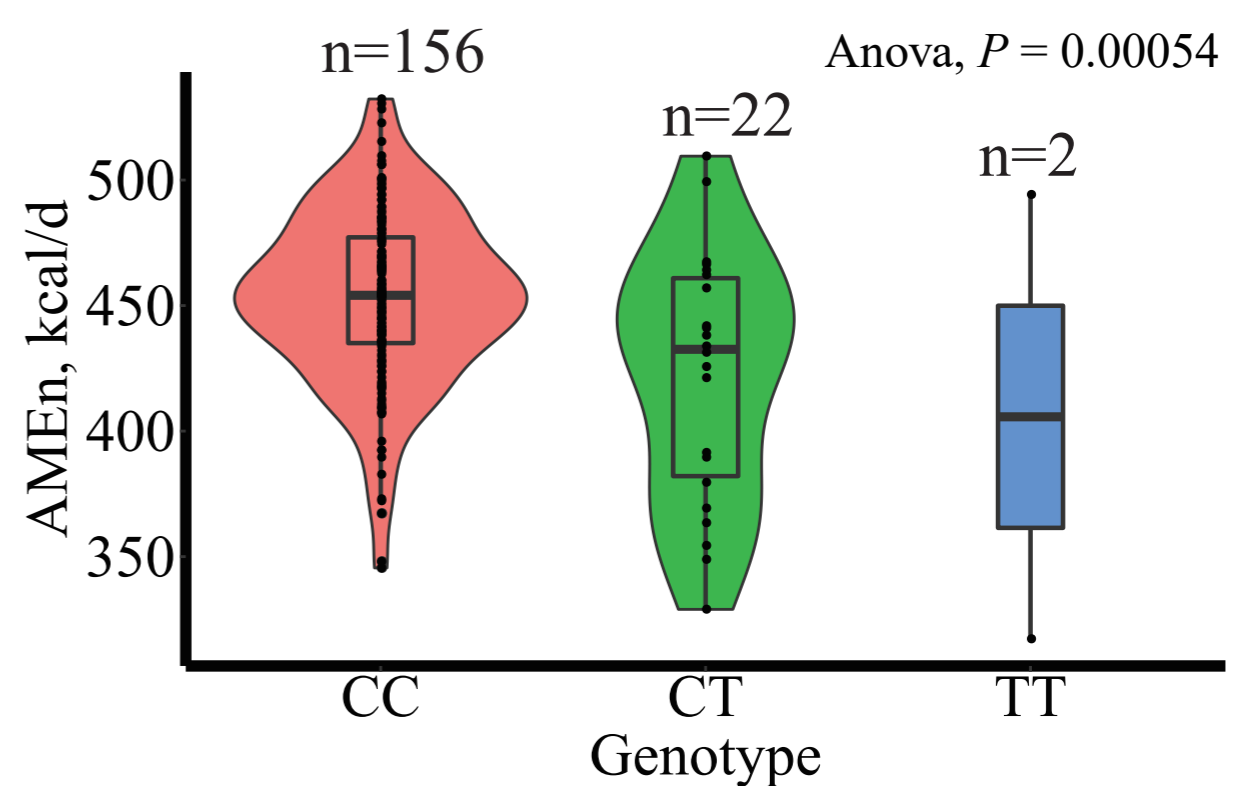

Supplement: Supplementary file 4 — Additional file 4: Figure S2. Association results of the candidate region on GGA1 (93.26–93.38 Mb) for MDM and AMEn. (a) Linkage disequilibrium (LD) analysis of the 4 significant SNPs on GGA1. (b) Box plot for the effect of the SNP rs732655996 on MDM. (c) Box plot for the effect of the SNP rs732655996 on AMEn. MDM and AMEn represent metabolizable dry matter and nitrogen corrected apparent metabolizable energy, respectively. [file 40104_2020_469_MOESM4_ESM.pdf]
