# Supplementary material for: New insights into the associations among feed efficiency, metabolizable efficiency traits and related QTL regions in broiler chickens
Source: J Anim Sci Biotechnol. 2020 Jun 26;11:65. doi: 10.1186/s40104-020-00469-8 (PMC7318453; doi:10.1186/s40104-020-00469-8)
Supplement: Supplementary file 2 — Additional file 2: Table S1. Distribution of SNPs used in the GWAS analyses after post-imputation filtering. [file 40104_2020_469_MOESM2_ESM.docx]

**Table S1** Distribution of SNPs used in the GWAS analyses after post-imputation filtering

| GGA^a^ | Physical Map, Mb^b^ | No. of SNP markers | Average distance, kb | R^2^ | Imputation accuracy |
| --- | --- | --- | --- | --- | --- |
| 1 | 197.61 | 336,274 | 0.588 | 0.938 | 0.924 |
| 2 | 149.68 | 180,516 | 0.829 | 0.937 | 0.921 |
| 3 | 110.84 | 133,770 | 0.829 | 0.935 | 0.924 |
| 4 | 91.32 | 139,616 | 0.654 | 0.936 | 0.923 |
| 5 | 59.81 | 59,904 | 0.998 | 0.936 | 0.927 |
| 6 | 36.37 | 38,193 | 0.952 | 0.935 | 0.935 |
| 7 | 36.74 | 37,257 | 0.986 | 0.933 | 0.922 |
| 8 | 30.22 | 29,771 | 1.015 | 0.937 | 0.927 |
| 9 | 24.15 | 31,444 | 0.768 | 0.937 | 0.921 |
| 10 | 21.12 | 38,116 | 0.554 | 0.935 | 0.926 |
| 11 | 20.2 | 45,018 | 0.449 | 0.936 | 0.919 |
| 12 | 20.39 | 53,910 | 0.378 | 0.937 | 0.923 |
| 13 | 19.17 | 23,333 | 0.822 | 0.933 | 0.925 |
| 14 | 16.22 | 26,972 | 0.601 | 0.938 | 0.925 |
| 15 | 13.06 | 16,060 | 0.813 | 0.944 | 0.940 |
| 17 | 10.76 | 12,336 | 0.872 | 0.938 | 0.925 |
| 18 | 11.37 | 15,949 | 0.713 | 0.940 | 0.935 |
| 19 | 10.32 | 15,332 | 0.673 | 0.943 | 0.920 |
| 20 | 13.9 | 19,575 | 0.710 | 0.940 | 0.929 |
| 21 | 6.84 | 6,881 | 0.994 | 0.939 | 0.931 |
| 22 | 5.46 | 1,378 | 3.962 | 0.934 | 0.933 |
| 23 | 6.15 | 1,783 | 3.449 | 0.936 | 0.930 |
| 24 | 6.49 | 7,376 | 0.880 | 0.935 | 0.925 |
| 25 | 3.98 | 127 | 31.339 | 0.964 | 0.938 |
| 26 | 6.06 | 2,728 | 2.221 | 0.936 | 0.922 |
| 27 | 8.08 | 1,326 | 6.094 | 0.939 | 0.948 |
| 28 | 5.12 | 4,401 | 1.163 | 0.940 | 0.927 |

^a^*Gallus gallus* chromosome.

^b^Physical length of the chromosome based on Gallus gallus-6.0.
