# Supplementary material for: New insights into the associations among feed efficiency, metabolizable efficiency traits and related QTL regions in broiler chickens
Source: J Anim Sci Biotechnol. 2020 Jun 26;11:65. doi: 10.1186/s40104-020-00469-8 (PMC7318453; doi:10.1186/s40104-020-00469-8)
Supplement: Supplementary file 3 — Additional file 3: Table S2. Pearson correlation coefficients for growth and metabolizable traits in broilers. [file 40104_2020_469_MOESM3_ESM.docx]

| Traits | ADFI | BW28 | BW42 | ADG | DEW | GEE | CPE | MDM | AMEn | MCP | coefficient  of MDM | coefficient  of AMEn | coefficient  of MCP | AbF | AbP |
| --- | --- | --- | --- | --- | --- | --- | --- | --- | --- | --- | --- | --- | --- | --- | --- |
| ADFI | 1.00 |  |  |  |  |  |  |  |  |  |  |  |  |  |  |
| BW28 | 0.41^**^ | 1.00 |  |  |  |  |  |  |  |  |  |  |  |  |  |
| BW42 | 0.87^**^ | 0.72^**^ | 1.00 |  |  |  |  |  |  |  |  |  |  |  |  |
| ADG | 0.90^**^ | 0.23^**^ | 0.85^**^ | 1.00 |  |  |  |  |  |  |  |  |  |  |  |
| DEW | 0.91^**^ | 0.36^**^ | 0.76^**^ | 0.79^**^ | 1.00 |  |  |  |  |  |  |  |  |  |  |
| GEE | 0.90^**^ | 0.36^**^ | 0.77^**^ | 0.79^**^ | 0.99^**^ | 1.00 |  |  |  |  |  |  |  |  |  |
| CPE | 0.83^**^ | 0.42^**^ | 0.69^**^ | 0.64^**^ | 0.90^**^ | 0.87^**^ | 1.00 |  |  |  |  |  |  |  |  |
| DDM | 0.98^**^ | 0.41^**^ | 0.87^**^ | 0.89^**^ | 0.83^**^ | 0.81^**^ | 0.76^**^ | 1.00 |  |  |  |  |  |  |  |
| AMEn | 0.99^**^ | 0.41^**^ | 0.86^**^ | 0.88^**^ | 0.84^**^ | 0.82^**^ | 0.79^**^ | 1.00^**^ | 1.00 |  |  |  |  |  |  |
| DCP | 0.88^**^ | 0.30^**^ | 0.79^**^ | 0.88^**^ | 0.68^**^ | 0.69^**^ | 0.47^**^ | 0.92^**^ | 0.90^**^ | 1.00 |  |  |  |  |  |
| coefficient of MDM | 0.00 | 0.03 | 0.07 | 0.07 | -0.41^**^ | -0.41^**^ | -0.33^**^ | 0.18^*^ | 0.14 | 0.29^**^ | 1.00 |  |  |  |  |
| coefficient of AMEn | 0.03 | 0.06 | 0.06 | 0.03 | -0.34^**^ | -0.40^**^ | -0.17^*^ | 0.19^*^ | 0.19^*^ | 0.20^**^ | 0.90^**^ | 1.00 |  |  |  |
| coefficient of MCP | -0.17^*^ | -0.21^**^ | -0.09 | 0.04 | -0.4^**^ | -0.36^**^ | -0.69^**^ | -0.07 | -0.12 | 0.31^**^ | 0.59^**^ | 0.32^**^ | 1.00 |  |  |
| AbF | 0.54^**^ | 0.24^**^ | 0.45^**^ | 0.44^**^ | 0.49^**^ | 0.48^**^ | 0.45^**^ | 0.53^**^ | 0.53^**^ | 0.47^**^ | 0.01 | 0.03 | -0.10 | 1.00 |  |
| AbP | 0.41^**^ | 0.11 | 0.28^**^ | 0.31^**^ | 0.38^**^ | 0.37^**^ | 0.35^**^ | 0.40^**^ | 0.41^**^ | 0.35^**^ | -0.01 | 0.02 | -0.09 | 0.98^**^ | 1.00 |

**Table S2** Pearson correlation coefficients for growth and metabolizable traits in broilers^1^

^1^ADFI, average daily feed intake; BW28, body weight at 28 d of age; BW42, body weight at 42 d of age; ADG, average daily gain; DEW, dry excreta weight; GEE, gross energy excretion; CPE, crude protein excretion; MDM, metabolizable dry matter; AMEn, nitrogen corrected apparent metabolizable energy; MCP, metabolizable crude protein; AbF, weight of abdominal fat; AbP, percentage of abdominal fat; **P* < 0.05, ***P* < 0.01.
