# Supplementary material for: New insights into the associations among feed efficiency, metabolizable efficiency traits and related QTL regions in broiler chickens
Source: J Anim Sci Biotechnol. 2020 Jun 26;11:65. doi: 10.1186/s40104-020-00469-8 (PMC7318453; doi:10.1186/s40104-020-00469-8)
Supplement: Supplementary file 5 — Additional file 5: Figure S3. Association results of the candidate region on GGA14 (10.02–10.71 Mb) for coefficient of MCP. (a) Linkage disequilibrium (LD) analysis of the 206 significant SNPs on GGA14. (b) Box plot for the effect of the SNP rs738484580 on the coefficient of MCP. Coefficient of MCP represents the coefficient of metabolizable crude protein. [file 40104_2020_469_MOESM5_ESM.pdf]

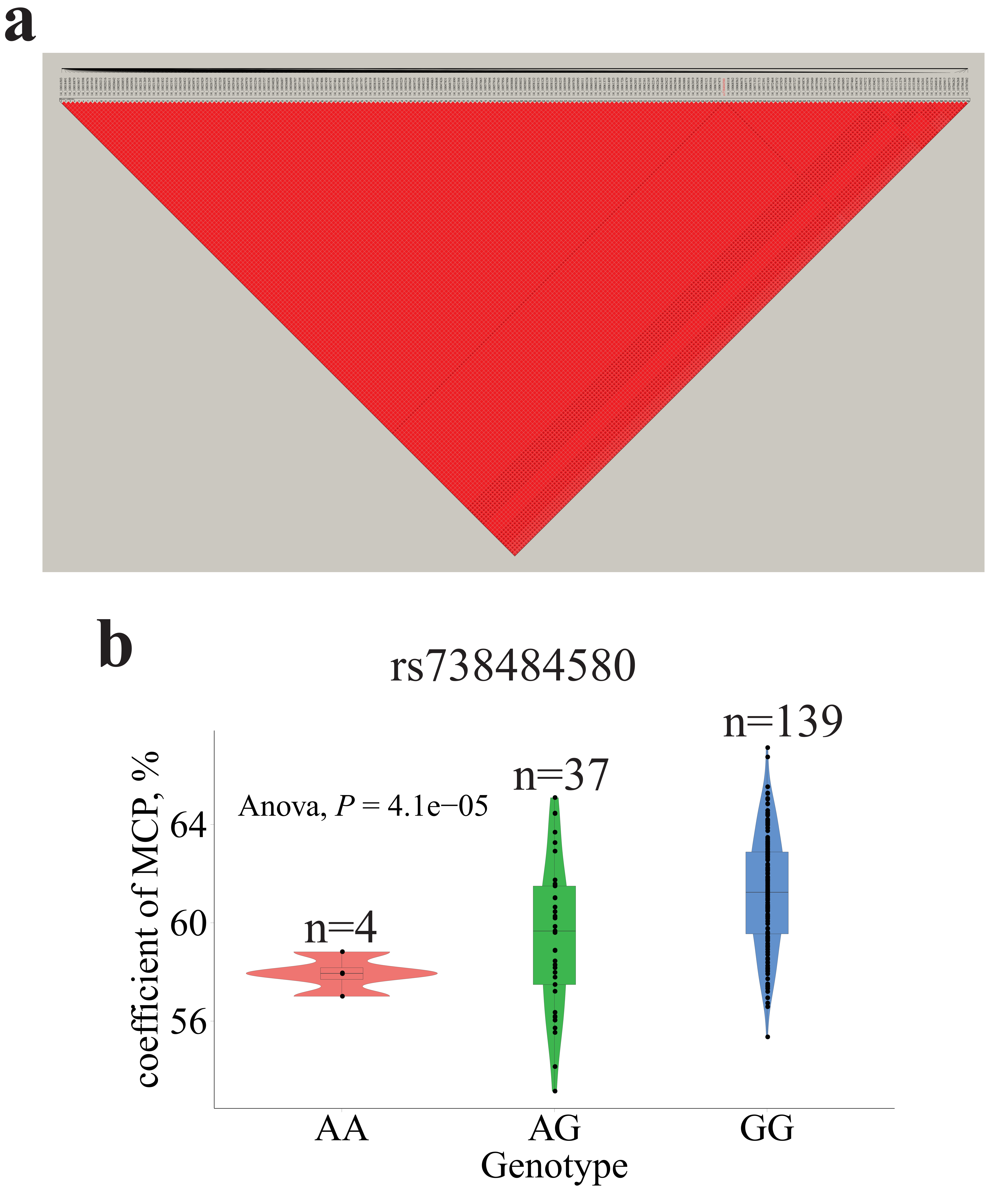
a
b
rs738484580
n=139
n=37
n=4
Anova, P = 4.1e−05
coefficient of MCP, %
64
60
56
AA
AG
GG
Genotype
